# The Short-Term Results of Autologous Platelet-Rich Plasma as an Adjuvant to Re-Intervention in the Treatment of Refractory Full-Thickness Macular Holes

**DOI:** 10.3390/jcm12052050

**Published:** 2023-03-04

**Authors:** Matilde Buzzi, Guglielmo Parisi, Paola Marolo, Francesco Gelormini, Mariantonia Ferrara, Raffaele Raimondi, Davide Allegrini, Tommaso Rossi, Michele Reibaldi, Mario R. Romano

**Affiliations:** 1 Department of Biomedical Sciences, Humanitas University, 20090 Milan, Italy; 2 Department of Surgical Sciences, Eye Clinic Section, University of Turin, 10124 Turin, Italy; 3 Manchester Royal Eye Hospital, Manchester University Hospitals NHS Foundation Trust, Manchester M13 9WL, UK; 4 Eye Unit, Department of Ophthalmology, Humanitas Gavazzeni-Castelli, 24125 Bergamo, Italy; 5 IRCCS Fondazione Bietti ONLUS, 00198 Roma, Italy

**Keywords:** autologous platelet-rich plasma, highly myopic full-thickness macular holes, optic disc pit maculopathy, pars plana vitrectomy, refractory full-thickness macular hole

## Abstract

The purpose of this study was to investigate the short-term efficacy and safety of autologous platelet-rich plasma (a-PRP) as an adjuvant to revisional vitrectomy for refractory full-thickness macular holes (rFTMHs). We conducted a prospective, non-randomized interventional study including patients with rFTMH after a pars plana vitrectomy (PPV) with internal limiting membrane peeling and gas tamponade. We included 28 eyes from 27 patients with rFTMHs: 12 rFTMHs in highly myopic eyes (axial length greater than 26.5 mm or a refractive error greater than -6D or both); 12 large rFTMHs (minimum hole width *>* 400 μm); and 4 rFTMHs secondary to the optic disc pit. All patients underwent 25-G PPV with a-PRP, a median time of 3.5 ± 1.8 months after the primary repair. At the six-month follow-up, the overall rFTMH closure rate was 92.9%, distributed as follows: 11 out of 12 eyes (91.7%) in the highly myopic group, 11 out of 12 eyes (91.7%) in the large rFTMH group, and 4 out of 4 eyes (100%) in the optic disc pit group. Median best-corrected visual acuity significantly improved in all groups, in particular from 1.00 (interquartile range: 0.85 to 1.30) to 0.70 (0.40 to 0.85) LogMAR in the highly myopic group (*p* = 0.016), from 0.90 (0.70 to 1.49) to 0.40 (0.35 to 0.70) LogMAR in the large rFTMH group (*p* = 0.005), and from 0.90 (0.75 to 1.00) to 0.50 (0.28 to 0.65) LogMAR in the optic disc pit group. No intraoperative or postoperative complications were reported. In conclusion, a-PRP can be an effective adjuvant to PPV in the management of rFTMHs.

## 1. Introduction

Pars plana vitrectomy (PPV) with an internal limiting membrane (ILM) peeling and gas tamponade is the current gold standard for the primary repair of full-thickness macular holes (FTMHs), leading to an overall reported closure rate of 80–100% [1,2]. Moreover, the use of an inverted ILM flap has been demonstrated to further increase the primary anatomical success rate in large or highly myopic (HM) or both FTMHs, that are at a higher risk of a primary failure [3,4]. However, the FTMHs that fail to close in the first instance, so-called refractory FTMH (rFTMH), still represent a surgical challenge and have been associated with a lower closure rate in the case of secondary repair [5]. To optimize the outcomes of rFTMH repair, a variety of surgical techniques involving revisional PPV combined with additional maneuvers or adjuvant tissues or both, has been proposed, such as a repeated gas tamponade with or without the enlargement of the previous ILM peeling, the application of subretinal fluid, a retinal massage, placement of a micro drain, relaxation of the arcuate or radial retinotomies, transplantation of the ILM-free flaps, construction of an autologous or allogenic lens capsular flap, use of a human amniotic membrane plug or autologous neurosensory retinal flap, outpatient treatment of fluid or gas exchange alone or in combination with laser photocoagulation and macular buckling [5]. So far, there is no agreement on the best surgical approach for rFTMH [6].

The use of autologous platelet-rich plasma (a-PRP) has also been suggested as an effective adjuvant to revisional PPV for the repair of rFTMH [7,8,9,10,11], based on its potential beneficial effects on retinal pigment epithelium (RPE) cells and Müller cells, as well as a potential contributing mechanical effect on MH closure [5]. Indeed, the a-PRP, consisting of a portion of the plasma obtained by the centrifugation of the peripheral blood, is characterized by a platelet concentration and, thus, growth factors (GFs) content, significantly higher compared with that of the original sample [12]. These GFs have been shown to exert a modulating effect on tissue inflammation as well as a promoting effect on tissue repair and regeneration [12]. Concerning the eye, several GFs contained in platelets have been associated with the modulation and promotion of migration and growth of Müller cells, which have an established crucial role in the healing process of the macular hole [12,13,14,15,16]. In particular, it has been demonstrated that the incubation of rat Müller cells with platelet-derived GF (PDGF), fibroblast GF, epidermal GF or insulin-like GF 1, resulted in the enhancement of the proliferative and migratory activity of these cells [17]. These findings confirmed previous experimental studies demonstrating a stimulating effect on immortalized Müller cell migration and the proliferation of different platelet preparations in vitro [15]. Concerning RPE cells, there is experimental evidence of enhanced RPE cell migration and proliferation in response to incubation with human thrombocyte concentrate [18]. Furthermore, PDGF has been specifically involved in the promotion of the proliferative and migratory activity of human RPE cells [18]. Concerning the potential mechanical effect of a-PRP, it has been speculated that the platelets coagulum could act by sealing the macular hole and, thus, contribute to its closure [19]. The evidence of a hyperreflective plug overlying the hole has been reported the day after the surgery using both a-PRP [20] and plasma rich in growth factor [19].

In this light, it may be hypothesized that rFTMH at a high failure risk, such as large or highly myopic holes or both, might benefit from the use of a-PRP to further promote their closure. The purpose of our study was to evaluate the efficacy, in terms of both visual and anatomic outcomes, and the safety of a-PRP as an adjuvant to revisional PPV in rFTMHs.

## 2. Materials and Methods

We conducted a prospective, nonrandomized, interventional case series on patients affected by rFTMH and treated with PPV with a-PRP between January 2021 and June 2022. The study was conducted per the tenets of the Declaration of Helsinki, Institutional review board approval was obtained (Protocol Number 0041666) and all patients signed a written informed consent form after a detailed discussion regarding the procedure.

### 2.1. Inclusion and Exclusion Criteria

The inclusion criteria were the following:

- A previous PPV with ILM peeling and gas tamponade due to an idiopathic FTMH or myopic FTMH or optic disc pit maculopathy (ODPM), and
- adult patients (age > 18 years), and
- rFTMHs associated with high myopia (defined as eyes with an axial length greater than 26.5 mm or a refractive error greater than -6D or both), or
- large rFTMHs (minimum hole width *>* 400 μm, according to the (OCT)-based International Vitreomacular Traction Study Group (IVTS) classification [21]), or
- rFTMHs associated with ODPM.

The exclusion criteria included previously vitrectomized eyes at the time of the PPV for an FTMH repair (in case of idiopathic or highly myopic FTMH) or ODPM, any concomitant ocular or neurological condition that could affect the visual acuity, uncontrolled systemic conditions potentially leading to an unacceptable increased operative risk, as well as uncontrolled or untreated ocular pathologies, or both, that were likely to result in a significant increase in the risk of intraoperative or postoperative complications or both.

### 2.2. Surgical Procedure

All patients underwent surgery under local anesthesia. The surgeries were performed by three experienced vitreoretinal surgeons. The primary surgery consisted of a three-port, trans-conjunctival, sutureless 25-gauge PPV. If needed, posterior vitreous detachment (PVD) was induced and a core and peripheral vitrectomy were carried out. A blue dye was used to stain the ILM and a conventional ILM peeling of at least a two-disc diameter was performed. A foveal-sparing ILM peeling was performed in the case of an ODPM. After the indented search to rule out any undetected peripheral pathology to treat, a fluid-air exchange (FAX) was performed and followed by an air-gas exchange. In all phakic patients, a concomitant standard small-incision cataract surgery was performed. All the patients were requested to keep facedown positioning for 3 days postoperatively.

Concerning the revisional surgical procedure, to prepare the a-PRP, immediately before the surgery, the patient’s peripheral venous blood was collected in a 10 mL tube with 1 mL of 3.2% sodium citrate, and centrifugated at 1600 revolutions per minute for 10 min. The PRP, identifiable as the middle of the 3 distinct visible layers (from the top; platelet-poor plasma, PRP, and red blood cells), was then collected in a sterile syringe.

The revisional PPV was performed using a 25-gauge PPV. The adequacy of the previous ILM peeling was checked after staining with a blue vital dye. None of the cases required an ILM peeling enlargement. The residual epiretinal traction, due to the initial foveal sparing, was removed from the eyes in the ODPM group. After FAX, 3 drops of aPRP were injected over the rFTMH and 12% perfluoropropane (C_3_F_8_) was used as tamponade. Finally, the patient was instructed to keep the supine position for 1 h, followed by the face-down position for 3 days.

### 2.3. Ophthalmic Evaluation

All of the patients were evaluated at a baseline, the day after the surgery and at a 1-, and 6-month follow-up (FU). At each FU, the patients underwent a complete ophthalmic examination, including a best-corrected visual acuity (BCVA) assessment, slit-lamp biomicroscopy, applanation tonometry, and dilated fundoscopy. In addition, spectral-domain optical coherence tomography (SD-OCT) of the macula was performed using the Heidelberg Spectralis SD-OCT (Heidelberg Engineering, Heidelberg, Germany) at the baseline and at the 1- and 6-month FU. A 30° × 25° posterior pole scan, 240 Sects., ART 20 was acquired for each patient and the crossline centered on the fovea was used to measure the hole diameter. According to the IVTS classification [21], the minimum hole width was manually drawing a line between the narrowest hole points at the level of the mid retina and parallel to the RPE, using the caliper function of the OCT device. According to the classification proposed by Rossi and coworkers [22], the MH closure pattern was classified as type 0 if the MH remained open with an exposed RPE, and type 1 in the case of MH closure with reconstitution of all of the retinal layers (1A), or with a residual defect of the external (1B) or internal (1C) retinal layers; as no autologous or heterologous tissue transplant was performed, the type 2 closure patter did not apply to this study. In addition, the presence of residual external limiting membrane (ELM) defects, ellipsoid zone (EZ) defects or fibrin-like hyperreflective (HR) tissue, or both, was documented. The closure pattern was classified as 1A if only the focal of the EZ and/or ELM were present.

### 2.4. Statistical Analysis

We carried out the statistical analysis using the IBM SPSS Statistics software (version 29; Armonk, NY, USA: IBM Corp.). As the visual acuity was measured in Snellen, the VA values were converted into logarithms of the minimal angle of resolution (logMAR) values for the statistical analysis. As previously performed in other studies, a logMAR value of 1.98, 2.28, 2.70, and 3.00 was considered equivalent to counting fingers, hand movements, perception of light, and no perception of light, respectively [23]. The BCVA values were expressed as a median and interquartile range. Before statistical analysis, the Shapiro-Wilk test was used to evaluate the continuous variables and, consequently, nonparametric statistical analyses were performed. The statistical significance of the differences between the median preoperative and postoperative BCVA was tested using the Wilcoxon signed-rank test for paired samples. The difference was considered statistically significant if the *p*-value <0.05.

## 3. Results

A number of 28 eyes, from 27 patients with rFTMH, were included in the study. Specifically, 12 eyes (42.9%) were highly myopic (HM group), 12 eyes (42.9%) had a large rFTMH, and 4 eyes (14.3%) (L group) had rFTMHs associated with ODPM (ODPM group). All the patients completed the minimum follow-up of 6 months. The mean patient age was 61 ± 8.78 years. The demographic findings are resumed in Table 1. The mean baseline FTMH size (before primary surgery) was 385.5 ± 148.5 μm in the HM group and 567.6 ± 118.7 μm in the L group. All eyes in the OPDM group presented initially with subretinal and intraretinal fluid, involving both inner and outer retinal layers, and developed FTMH after a primary PPV and ILM peeling. The mean interval between the first and the second surgical procedure was 3.5 ± 1.8 months.

The mean macular hole size before the revisional PPV was 451.8 ± 162.4 μm in the whole cohort, and, specifically, 364 ± 147.7 μm in the HM group, 552.4 ± 145.4 μm in the L group, and 413.5 ± 79.9 μm in the ODPM group.

**Figure 1:**
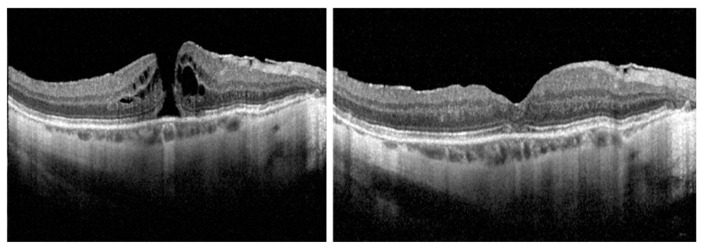
Case 1: A full-thickness refractory macular hole (rFTMH) of a highly myopic eye that did not close after a primary 25-G pars plana vitrectomy (PPV) with internal limiting membrane peeling (**left**). At 6 months after secondary PPV with autologous platelet-rich plasma and gas tamponade, complete closure of rFTMH is seen (**right**).

**Figure 2:**
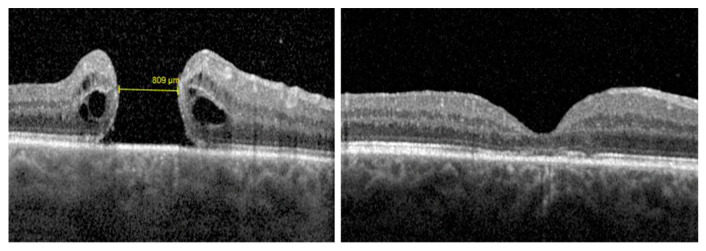
Case 2: A full-thickness refractory large macular hole (rFTMH) (minimum hole width 809 μm) that did not close after a primary 25-G pars plana vitrectomy (PPV) with internal limiting membrane peeling (**left**). At 6 months after secondary PPV with autologous platelet-rich plasma and gas tamponade, complete closure of rFTMH is seen (**right**).

**Figure 3:**
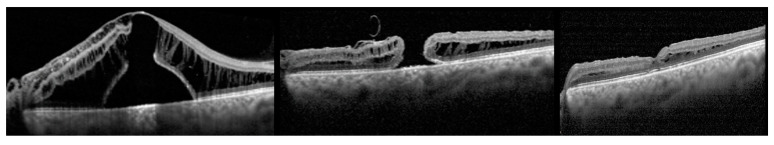
Case 3: An optic disc pit maculopathy with intraretinal fluid involving inner and outer retinal layers and subretinal fluid (**left**). A secondary full-thickness refractory macular hole after a primary 25-G pars plana vitrectomy (PPV) with internal limiting membrane peeling and foveal sparing (**middle**). The FTMH was closed at 3 months after the secondary PPV with autologous platelet-rich plasma and gas tamponade (**right**).

At the 6-month FU, hole closure was achieved in 26 out of 28 eyes (92.9%): 11 out of 12 eyes in the HM group (91.7%), 11 out of 12 eyes in the L group (91.7%) and 4 out of 4 eyes in the ODPM group (100%). The structural OCT outcomes at the 6-month FU are shown in Table 1. Complete reconstitution of all the retinal layers was detected in the majority of the rFTMHs as a type 1A closure pattern and was documented in 46.4% of the eyes, with the highest percentage in the ODPM group (75%). Residual EZ defects resulted to be more commonly detected than residual ELM defects, regardless of the initial type of rFTMH. Indeed, this trend was noted in all the groups, with the highest rate of residual EZ defect in the L group (Table 2). Larger FTMH showed a trend towards the residual EZ defect as the mean preoperative hole size was 465.4 ± 180.6 μm and 423.2 ± 119.3 μm in eyes with and without the residual postoperative EZ defect, respectively. Two representative cases are shown in Figure 1, Figure 2 and Figure 3.

The median overall preoperative BCVA was 0.95 (0.75 to 1.15) LogMAR. In particular, the median preoperative BCVA was 1.00 (0.85 to 1.30) LogMAR in the highly myopic group, 0.90 (0.70 to 1.49) LogMAR in the large rFTMH group, and 0.90 (0.75 to 1.00) LogMAR in the optic disc pit group. At the final 6-month FU, a statistically significant improvement in BCVA was documented in both the whole cohort and each of the groups identified. In particular, the median BCVA improved from 0.95 (0.75 to 1.15) LogMAR to 0.50 (0.40 to 0.70) LogMAR in the whole cohort (*p* < 0.001), 1.00 (0.85 to 1.30) LogMAR to 0.70 (0.40 to 0.85) LogMAR in the highly myopic group (*p* = 0.016), and from 0.90 (0.70 to 1.49) LogMAR to 0.40 (0.35 to 0.70) LogMAR in the large rFTMH group (*p* = 0.005). The median BCVA improved from 0.90 (0.75 to 1.00) LogMAR to 0.50 (0.28 to 0.65) LogMAR in the ODPM group; however, the size of the sample was too small to be tested for statistical significance.

No intraoperative or postoperative complications were recorded.

ELM, external limiting membrane; EZ, ellipsoid zone; HR, hyperreflective; ODPM, optic disc pit maculopathy; rFTMHs, refractory full-thickness macular holes.

## 4. Discussion

Pars plana vitrectomy with ILM peeling and gas tamponade currently represents the established treatment of choice for the primary repair of idiopathic FTMH; in addition, the inverted ILM flap is gaining popularity for the primary repair of large or highly myopic FTMH, or both, as it has been associated with an increased closure rate in these types of FTMH, that are at higher risk of failure [1,24]. Unsuccessful closures of FTMH after primary surgical repair occurs in up to 10% of cases and there is currently no consensus on the best surgical management [5]. Performing a revisional PPV for rFTMH involves some specific challenges associated with the absence or limited availability of ILM in the macular area and a decreased rate of hole closure [25]. In addition, a higher risk of primary and secondary failure as well as poorer visual outcomes is known to be associated with large or highly myopic FTMH or both [5]. Refractory FTMHs associated with OPDM after the primary surgery can represent a surgical scenario similar to rFTMH after surgical repair for the idiopathic FTMH, as PPV and ILM peeling with or without an ILM inverted flap are gaining a growing popularity as the primary surgical approach in treating OPDM [26]. So far, various revisional surgical techniques have been proposed for the treatment of rFMTHs, but the strength of the evidence based on the available studies is limited by several important flaws, such as the predominance of retrospective studies, the absence of randomized controlled trials, the variety of methods used, and the surgical steps described [5]. Despite the above-mentioned limitations, the literature appears to support the combination of revisional PPV with the use of adjuvants aimed at the promotion and modulation of the intraretinal gliosis or the mechanical action of “scaffold” for the Müller cells or both [5].

In light of its high GF content, a-PRP has been long used to promote tissue regeneration or repair in different fields of medicine, including ophthalmology [12]. In this regard, PRP has been first and successfully used in the management of diseases of the ocular surface [27]. The application of a-PRP as an adjuvant in FTMH surgery has been supported by the experimental evidence of the stimulatory effect of platelet GFs on the migratory and proliferative activity of Müller cells, as well as RPE cell growth in vitro [11,13,17]. So far, the use of a-PRP has been associated with promising results in terms of the closure rate and visual gain in macular holes of different types, such as highly myopic FTMH [19], idiopathic large FTMH [28], rFTMH associated with Alport syndrome [20], FTMH associated with macular telangiectasia type 2 [29], lamellar macular holes [30], idiopathic rFTMH associated, or not associated, with high myopia [5]. However, it is worth noting that the strength of these findings is limited by the availability of only a few retrospective studies [5]. So far, only one randomized controlled trial compared the outcomes of PPV and ILM peeling with or without an intraoperative injection of autologous platelet concentrate (APC) in the eyes affected by recurrent FTMHs, highly myopic FTMHs, or large MHs [28]. Despite the inter-group difference in the closure rate not reaching statistical significance, the use of APC was associated with a trend towards a higher success rate compared with PPV and ILM peeling only, and so, it was suggested as a potential adjuvant in selected cases [28].

In this prospective study, we evaluated the efficacy and safety of a-PRP as an adjuvant to revisional PPV for rFTMHs at high risk of failure, such as large rFTMHs, highly myopic rFMTH, and rFTMHs associated with OPDM. Out of a total of 28 eyes included, a type 1 closure rate was achieved in 92.9% of cases (26 eyes), with one large and one highly myopic rFTMH that failed to close. These results are consistent with those of Figueroa et al. [19], who reported a successful hole closure in 10 of 11 highly myopic rFTMHs treated with revisional PPV and plasma rich in growth factors, a subtype of aPRP that need to be activated before the surgical use. The closure rate reported in the studies currently available on the use of a-PRP in rFTMH repair ranges from 60% to 85% [10,25,28,31,32]. The short median inter-surgery interval (3.5 ± 1.8 months) may have contributed to the high closure rate in our study, as a shorter time between the primary and secondary surgery may result in a higher anatomical success rate [11]. In addition, Degenhardt et al. [11] evaluated the outcomes of 103 eyes treated with revisional PPV and autologous platelet concentrate due to rFTMH and reported that there was a trend towards the correlation of greater axial length and a higher rate of anatomical failure. However, in this study, the closure rate was high (at 91.7%) in both the HM group and the L group, with only one hole that failed to close in each group. Finally, the use of a-PRP in revisional PPV has been associated with ILM peeling enlargement, if needed, and a different intraocular tamponade, including short-acting gas, long-acting gases and silicone oil [5]. In this regard, an additional strength of our study is the absence of the need for ILM peeling enlargement and the use of the same gas tamponade (C_3_F_8_) in all surgeries. Indeed, this ruled out the potential effect of the ILM peeling enlargement or different intraocular tamponade, or both, on the final closure rate.

In terms of the anatomical results, we also analyzed the restoration of EZ and ELM. It is known that ELM recovery is more common in the EZ recovery in eyes treated for FTMH [33]. The recovery of ELM and EZ has been previously correlated with better visual outcomes [34]. In our study, we observed a complete restoration of the ELM in the majority of the eyes (74.4%), whereas residual EZ defects were detected in 75% of the eyes at the 6-month FU. This finding appears consistent with the residual EZ damage in 76% and 81% of eyes treated for rFTMH previously reported by Purtskhvanidze et al. [10] and Degenhardt et al. [11], respectively. It has been speculated that the greater hole size may be correlated with a higher rate of residual EZ and ELM damage [19]; thus, the large size of the rFTMHs at the baseline may have contributed to the presence of residual EZ damage in our sample. Degenhardt et al. [11] also hypothesized that the EZ damage might be considered a potential complication of a-PRP. Nevertheless, as highlighted by the same authors [11], persistent damage of the EZ band is a known complication of the FTMH surgery itself and so far, there is no evidence supporting a specific role of a-PRP.

Another important structural complication potentially associated with the surgical repair of FTMH is the postoperative evidence of excessive intraretinal gliosis, which has been associated with worse reconstitutions of the ELM and EZ, as well as poorer visual outcomes due to a detrimental effect in retinal neuronal cells [35]. A potential beneficial effect of PRP in terms of the alleviation of the fibrotic reaction has been demonstrated in the rat model of dimethylnitrosamine-induced hepatic fibrosis [36]. This may support a modulating effect of platelet GFs in wound-healing processes, such as intraretinal gliosis. This potential beneficial effect of a-PRP might be also more important in eyes that may be at a higher risk of excessive intraretinal gliosis, such as highly myopic eyes. Consistent with this hypothesis, we detected HR fibrin-like tissue only in one highly myopic eye.

Concerning the functional results, we reported a significant improvement in BCVA from the baseline to the final FU visit, in all groups. The potential advantages of revisional PPV with aPRP compared to other revisional surgical techniques for rFTMHs, in terms of final VA and VA gain, have been recently highlighted. Indeed, in a recent retrospective multicentric study comparing several revisional surgical techniques for rFTMHs, revisional PPV and a-PRP were associated with the highest visual gain (a mean of 24 ETDRS letters gain, ranging from 12 to 38 letters) compared to revisional PPV with repeating gas tamponade, ILM-free flap, radial nerve fiber layer incisions, retinal massage, and the fitting of a micro drain [37]. In addition, a recent review comparing anatomical and functional outcomes of different surgical revisional techniques for treating rFTMHs concluded that revisional PPV with a-PRP represents one of the most efficient techniques available in light of the good anatomical and functional outcomes and the low level of complexity of surgical maneuvers [27]. In particular, Frisina et al. [38] confirmed the superiority of a-PRP in terms of the BCVA gain when compared with ILM-free flap transplantation and pointed out that the only surgical technique associated with BCVA gain greater than a-PRP was the use of a human amniotic membrane plug, that entails more invasive and challenging surgical maneuvers.

Finally, it is worth highlighting that additional advantages of a-PRP lie in its simplicity and safety. The collection and delivery protocol of a-PPR is minimally invasive, rapid, and repeatable. As briefly mentioned above, concerning the comparison with the use of a human amniotic membrane plug, this advantage of a-PRP is also more important if compared with the more complex surgical maneuvers of other validated revisional techniques, such as ILM-free flap transplantation, or the greater invasiveness, or both, for instance in the case of autologous retinal free flap transplantation. In addition, no intraoperative and postoperative complications were recorded. Although a theoretical risk of an increased risk of endophthalmitis and severe intraocular inflammation associated with the intraocular use of a-PRP has been raised in the past [9,39], this concern is not supported by any available evidence. Indeed, to the best of our knowledge, no case of postoperative endophthalmitis has ever been reported in eyes treated with PPV and different formulations of platelet concentrate. In addition, a recent retrospective case series that compared eyes with rFTMH treated with revisional PPV and heavy silicone oil versus revisional PPV with autologous platelets concentrate and SF_6_, reported severe postoperative complications (namely endophthalmitis and retinal detachment associated with proliferative vitreoretinopathy) only in the former group, supporting the safety of a-PRP use [40]. A case has been described of temporary and self-resolved exudative retinal detachment during the second week after PPV with platelet concentrate and without ILM peeling [39]; in this case, the potential causative role of the platelet concentrate or the unusually high concentration of inflammatory mediators due to an unusual white cells/platelets breakdown in the concentrate, or both, has been speculated but no evidence supporting this hypothesis has been presented [39]. No other case of postoperative complications induced by severe intraocular inflammation has been reported in the literature, so far. Consistently, none of the patients included in this study experienced any infection or excessive intraocular inflammation.

We acknowledge that the small sample is a limitation of this study. However, we specifically focused on subgroups of rFTMH that are known to be at higher risk of failure and, differently to the currently available studies, we presented a study with a prospective design. In addition, future studies could analyze more detailed functional outcomes, such as metamorphopsia and retinal sensitivity. Finally, we did not compare the use of a-PRP in revisional PPV for rFTMH with alternative revisional techniques and we did not include a control group treated with revisional PPV and repeated gas tamponade alone. This analysis could be carried out in future larger prospective studies, ideally randomized.

In conclusion, revisional PPV with a-PRP can be an effective and safe treatment for rFTMHs, resulting in satisfactory visual and anatomical outcomes comparable with other surgical options and the advantage of a simple and reproducible procedure.

## Figures and Tables

**Table 1:** The demographic and clinical findings.

	Overall	HM Group	L Group	ODPM Group
Age (mean, years)	61 ± 8.78	62.1 ± 6.6	65 ± 6.3	46 ± 4.5
Sex (% male)	42.8%	33.3%	41.7%	75%
Laterality (% right)	53.6%	66.7%	50%	25%

**Table 2:** The optical coherence tomography data at 6-month follow-up.

	Overall	Highly Myopic rFTMHs	Large rFTMHs	rFTMHs Associated with ODPM
Closure rate	26/28 (92.9%)	11/12 (91.7%)	11/12 (91.7%)	4/4 (100%)
Closure type 1A	13/28 (46.4%)	5/12 (41.7%)	5/12 (41.7%)	3/4 (75.0%)
Closure type 1B	2/28 (7.1%)	1/12 (8.3%)	0/12 (0%)	1/4 (25.0%)
Closure type 1C	11/28 (39.3%)	5/12 (41.7%)	6/12 (50.0%)	0/4 (0%)
Residual ELM defect	8/28 (25.6%)	6/12 (50.0%)	4/12 (33.3%)	1/4 (25%)
Residual EZ defect	21/28 (75.0%)	8/12 (66.7%)	11/12 (91.7%)	2/4 (50%)
Fibrin-like HR tissue	1/28 (3.6%)	1/12 (8.3%)	0/12 (0%)	0/4 (0%)

## Data Availability

The data that support the findings of this study are available from the corresponding author upon reasonable request.
